# A qualitative assessment of nonclinical drivers of pediatric outpatient antibiotic prescribing: The importance of continuity

**DOI:** 10.1017/ash.2022.224

**Published:** 2022-06-29

**Authors:** Hillary J. J. Spencer, Sophie Katz, Milner Staub, Carolyn M. Audet, Ritu Banerjee

**Affiliations:** 1 Division of Infectious Diseases, Department of Pediatrics, Vanderbilt University Medical Center, Nashville, Tennessee; 2 Division of Infectious Diseases, Department of Medicine, Vanderbilt University Medical Center, Nashville, Tennessee; 3 Department of Health Policy, Vanderbilt University Medical Center, Nashville, Tennessee

## Abstract

**Background and objectives:**

Antibiotic overuse is common in outpatient pediatrics and varies across clinical setting and clinician type. We sought to identify social, behavioral, and environmental drivers of outpatient antibiotic prescribing for pediatric patients.

**Methods:**

We conducted semistructured interviews with physicians and advanced practice providers (APPs) across diverse outpatient settings including pediatric primary, urgent, and retail care. We used the grounded theory constant comparative method and a thematic approach to analysis. We developed a conceptual model, building on domains of continuity to map common themes and their relationships within the healthcare system.

**Results:**

We interviewed 55 physicians and APPs. Clinicians across all settings prioritized provision of guideline-concordant care but implemented these guidelines with varying degrees of success. The provision of guideline-concordant care was influenced by the patient–clinician relationship and patient or parent expectations (relational continuity); the clinician’s access to patient clinical history (informational continuity); and the consistency of care delivered (management continuity). No difference in described themes was determined by setting or clinician type; however, clinicians in primary care described having more reliable relational and informational continuity.

**Conclusions:**

Clinicians described the absence of long-term relationships (relational continuity) and lack of availability of prior clinical history (informational continuity) as factors that may influence outpatient antibiotic prescribing. Guideline-concordant outpatient antibiotic prescribing was facilitated by consistent practice across settings (management continuity) and the presence of relational and informational continuity, which are common only in primary care. Management continuity may be more modifiable than informational and relational continuity and thus a focus for outpatient stewardship programs.

Antimicrobial overuse is common^1,2^ despite adverse consequences including antimicrobial resistance,^3^ excess cost,^4^ adverse reactions,^5^ and microbiome disruption.^6^ In 2013, 67 million outpatient antibiotic prescriptions were dispensed to children in the United States, or 813 antibiotic prescriptions per 1,000 children.^7^ Approximately 40% of antibiotic prescriptions to patients of all ages are written from urgent care and retail clinics.^8^ Although it is estimated that 30% of these prescriptions are inappropriate,^1^ antibiotic prescribing appropriateness has been shown to vary by clinical setting.^8^

In the past decade, there has been increasing recognition of prescribing as a social and behavioral phenomenon in addition to a clinical act.^9^ Many nonclinical factors have been described as drivers of inappropriate antimicrobial prescribing, including time pressures, belief that antibiotics are overprescribed, actual or perceived patient desire for antibiotics, age of the patient, age or experience of the clinician, perception of social responsibility, risk tolerance, and perception of applicability of treatment guidelines.^9–12^

Few studies have evaluated clinician perception of nonclinical drivers of antibiotic prescribing, only 2 studies were from the United States,^13,14^ and few included clinicians in urgent and retail care clinics or advanced practice providers (APP). Incorporating perceptions of clinicians in urgent and retail care clinics is important given their large proportion of antibiotic prescribing.^8^

In this study, we sought to describe and compare clinicians’ perceptions of nonclinical drivers of outpatient antibiotic prescribing for pediatric patients across diverse ambulatory settings. We evaluated the data using an existing framework for domains of continuity including relational, informational, and management continuity as described by Haggerty et al (Table 1).^15^

**Table 1.** Three Types of Continuity (as described by Haggerty et al)^15^

Domain of Continuity	Description
Informational continuity	The use of information on past events and personal circumstances to make current care appropriate for each individual (ie, access to records about what has occurred previously for a patient)
Management continuity	A consistent and coherent approach to the management of a health condition that is responsive to a patient’s changing needs (ie, a standardized approach to diagnosis and management including guideline-concordant care if appropriate)
Relational continuity	An ongoing therapeutic relationship between a patient and 1 or more clinicians

## Methods

### Setting

Participants were attending physicians or advanced practice providers (nurse practitioners or physician assistants) who care for children and were working in practices affiliated with Vanderbilt University Medical Center. Practice settings included pediatric primary care (pediatricians and APPs), retail health (APPs only), and urgent-care clinics including walk-in clinics that care for adult and pediatric patients (ie, APPs and family medicine, internal medicine, and medicine–pediatrics physicians) and pediatric-only clinics (ie, pediatricians only).

### Sampling strategy and ethical issues

All clinicians from included settings were invited to participate through e-mail. Recruitment continued until representative sampling from each setting and clinician type (physician and advanced practice provider), and thematic saturation were achieved. Interviews were conducted in person or by telephone. This project was approved by the Vanderbilt University Institutional Review Board with a waiver of informed consent

### Interviews

Interviews included open-ended questions to elicit participant experiences with prescribing antibiotics for children in ambulatory settings and factors that influenced those experiences. An interview guide was developed through a process of literature review, consultation with content and methodological experts, and pilot testing for length and comprehensibility. Questions addressed prescribing priorities and general considerations, modifying social and behavioral features, discussion of sample cases (selected to be ambiguous relative to guidelines), and questions about unique factors related to care settings (see the interview guide in the Supplementary Material). All interviews were conducted and analyzed by a single researcher (H.S.).

### Data analysis and techniques to enhance trustworthiness

Interviews were recorded and transcribed verbatim. Demographic data were stored in the REDCap database (Vanderbilt University, Nashville, TN). Transcripts were uploaded into MAXQDA qualitative data software (VERBI Software, 2020, Berlin, Germany) for management and analysis.

Data analysis was based on the grounded theory constant comparative method^16^ and a thematic analysis approach. Using ResearchTalk’s Sort and Sift, Think and Shift methodology,^17^ an interim analysis of 10 representative interviews identified preliminary categories of data to inform a code system, was applied to the entire data set. After all transcripts were coded, the code system was refined to organize analysis around domains of continuity^15^ based on the emergence of concepts suggested by the data. The full data set was verified with the final code set. Results were organized into a conceptual model to visualize relationships between the domains of continuity and factors influencing each domain.

Trustworthiness was enhanced through iterative questioning and data saturation. Additionally, results were presented to a subset of participants in a member-checking activity conducted by teleconference (n = 10) or e-mail (n = 2).

## Results

In total, 55 clinicians were interviewed: 17 (31%) from primary care, 13 (24%) from retail health, and 25 (45%) from urgent-care clinics (Table 2). Furthermore, 28 (51%) were APPs and 27 (49%) were physicians. Overall, 45 interviews (82%) were conducted by telephone and 10 (18%) were conducted in person. We found no difference in identified themes by setting or clinician type; however, clinicians in primary care described having more reliable relational and informational continuity.

**Table 2.** Characteristics of Participants

Characteristic	Total Participants (n = 55), No. (%)
**Clinician type**	
Physician	27 (49)
Advanced practice provider	28 (51)
**Setting**	
Primary care	17 (31)
Retail clinic	13 (24)
Urgent care	25 (45)
**Full time vs not full time (per diem or part-time) in setting**	
Full time	44 (80)
Not full time	11 (20)
**Sex**	
Female	43 (78)
Male	12 (22)
**Experience**	
0–5 y	13 (24)
6–10 y	13 (24)
11–15 y	13 (24)
16–20 y	12 (22)
21–25 y	3 (5)
26–30 y	0
>30 y	1 (2)
**Age group**	
20–30 y	4 (7)
31–40 y	22 (40)
41–50 y	22 (40)
51–60 y	7 (13)

### Conceptual model of nonclinical factors

**Fig. 1.**
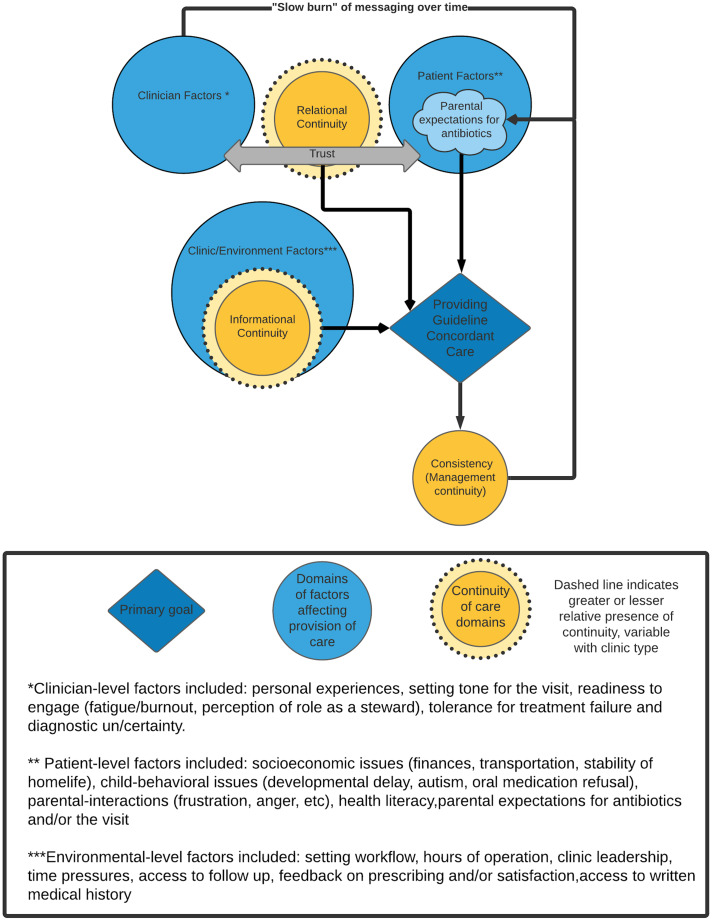
Conceptual model of relationships between primary nonclinical drivers of outpatient antimicrobial prescribing for pediatric patients.

At the model’s center is the theme “providing guideline concordant care” (Fig. 1). Participants described the desire to prescribe in accordance with guidelines as their primary priority. The ability to do so was supported by the presence of relational, informational, and management continuity and made more difficult by their absence. Participants also identified a variety of nonclinical influences across care settings, such as social determinants of health (finances, transportation, parental employment, stability of homelife), health literacy, setting workflow, hours of operation, tolerance for risk of treatment failure, diagnostic uncertainty, and clinician readiness to engage. These features were categorized into domains of clinician, patient, and environmental factors.

### Central priority: Guideline-concordant care

Most clinicians identified “guideline recommended” as the primary factor informing their prescribing decisions for pediatric patients. Guideline-concordant care was important for several reasons. Guidelines incorporate evidence and expert opinion, which some clinicians cited as improving confidence in their treatment recommendation. Guidelines also incorporate new knowledge, antibiotic susceptibilities, and antibiotic resistance patterns:

*“I believe in the importance of using research and empiric evidence to support clinical practice. I also don’t have a lot of confidence in my own memory of my education or of the longevity of the lessons I received 10, 15, 20 years ago in medical school. Knowledge changes. Available medications change. Antibiograms change. People change. And even for things I treat regularly, the knowledge around them does change. And so, I think it serves our patients better to provide treatments that are more aligned with the best available evidence.”—Physician, urgent care*

### Theme of management continuity

Clinicians across settings relayed the importance of having consistent management practices (management continuity). Consistency in management was described as being important within an individual’s practice, within a clinical setting (ie, among all clinicians in an specific clinic), and across settings (ie, across a healthcare system).

*“I feel like we all need to be on the same team, too. I think [the institution] does a great job of that, but it’s frustrating when I hear of patients who go somewhere else and get a Rocephin shot and an antibiotic for their cold. … I just want all the providers across the U.S. and the world, even, let’s just all practice within these guidelines. Because it’s such a big deal.”—*APP, retail clinic

In all clinical settings, participants shared the perception that previous antibiotic experiences were the primary influence on parental expectations for antibiotics, reflecting the presence or absence of management continuity. Previous experiences with antibiotics may promote expectations for antibiotics, but they may also shape expectations in a way that enables guideline concordant care if previous care did not involve an unnecessary antibiotic. Previous parental experiences were perceived by participants as not limited to the child for whom they were currently seeking care, but also included experiences for other children and for themselves, either historically or for a concurrent illness in the adult.

*“I definitely feel like it’s confusing for families because the adults often have… very similar symptoms as the child. They’ll go to some sort of walk-in urgent care or their regular place that they see a provider and they’ll get an antibiotic, and they’ll say, ‘I’m being treated for bronchitis or whatever, and I have this antibiotic and I have this steroid. And I think my kid [has] got the same thing.’”*—Physician, urgent care

### Theme of relational continuity

Relational dynamics and relational continuity were described by clinicians in all settings and were influenced by patient and clinician factors. For pediatricians in urgent care specifically, the importance of guidelines in fostering consistency of care across the healthcare system was one way to balance the absence of relational continuity. However, clinicians who commonly had a longstanding relationship with their patients, like those in primary care, were more likely to describe parent expectations that were already aligned with the clinicians’ practice.

*“It’s easier, of course, if I’m the PCP [primary care physician] because you already have that relationship with the family, the trust and confidence and so it’s easier to work through that conversation.”*—Physician, primary care

Parental expectations were also thought to be shaped by a “slow burn” of messaging over time. This theme was shared by a portion of participants who described the process of bringing parental expectations into alignment with guidelines as happening over time rather than being a one-time event. This highlighted the relationship between relational continuity and management consistency. Consistent practice can shape parental expectations for antibiotics and thus facilitate guideline-concordant prescribing. Consistency was perceived to facilitate trust in a clinician when the clinician does not have an existing relationship with the patient.

*“As things change slowly and more and more people do or don’t [do] things, it just becomes more normalized…. it just takes [a] slow burn.”—*Physician, urgent care

In addition to having better aligned expectations with a known patient via management continuity, a pre-existing relationship between the clinician and patient was described as facilitating guideline concordant care through mutual trust. When relational continuity was absent, clinicians described having to foster a parent’s trust de novo. They did this through spending time (to explain their reasoning, to allay fears, etc), through shared decision making, by exuding confidence, and by providing access to information about their decision making. Trust in the clinician was also perceived to be improved when management continuity exists. Relational continuity was also described as facilitating knowledge of the patient’s history and family dynamics (informational continuity). Such a relationship was described only as routinely existing in the primary-care setting and even there did not always exist.

A patient–clinician relationship is bidirectional, and clinicians must trust a patient as well as gain their trust. When relational continuity does not exist, clinicians must de novo develop trust in the patient or parent. One way clinicians may do this is by drawing on documentation of previous encounters if they have access to a patient’s medical record (informational continuity).

*“I look at a patient’s chart before I go in there and then I kind of see this patient has gone to the emergency six times in the last 2 months for fever for 12 hours. So you can kind of … not that I prejudge but I kind of do look at how often they bring their child in… [and] I make sure to document in my chart if a parent voices their dissatisfaction with my service of not giving them an antibiotic. It’s helpful for me because I would want to know.”—*APP, primary care

### Theme of informational continuity

In addition to informing a clinician of a patient’s trustworthiness with regard to historical care seeking and compliance with recommendations, informational continuity was described as informative to a clinician’s medical decision making. The decision to prescribe antibiotics was perceived to be informed by a patient’s past medical history including frequency of previous infections, length of time since last antibiotic prescription, and most recent class of antibiotic exposure. Although parents may provide a history, clinicians described that a verbal history may lack sufficient details or accuracy.

*“‘Oh, but my kiddo is prone to getting strep. They get strep all the time,’ but when I take the time to really look that over with them, sometimes they’re surprised…. The timeline blurs and when I take that extra time to go over that data with them about their specific child, because sometimes it crosses, the sibling had strep and it wasn’t this child.”*—Pediatrician, primary care

The availability of information feedback after the visit (post visit informational continuity) varied across settings. Clinicians in non–primary-care settings described the difficulty making contingency plans because of poor informational continuity post visit and limited or no feedback about the ultimate outcomes for their patient. Clinicians in primary care, on the other hand, described the benefit of often having longitudinal information on their patients.

*“I honestly don’t know how often [antibiotic failure] happens because a lot of our patients follow up with a lot with other providers because our clinic has such weird hours and so many providers. So maybe that’s occurring more than individual providers know because they follow up with somebody else a couple days later. So we don’t know that the antibiotic didn’t work.”—*APP, primary care

Informational continuity was described as only reliably (but not uniformly) existing in the primary care setting. Informational continuity variably existed in other urgent- and retail-care settings when patients have previously been seen at in-network clinics that share medical records.

## Discussion

By interviewing clinicians in diverse ambulatory settings, we identified practitioner values and perceptions of nonclinical influences on antibiotic prescribing for pediatric patients. We identified 3 domains of continuity that clinicians felt were essential for the provision of guideline concordant care: relational continuity, informational continuity, and management continuity.^15^ Additionally, we developed a conceptual model to visualize how these themes are related and how patient, clinician, and environmental factors affect these themes.

In our study, both physicians and APPs across settings placed a high value on guidelines and prioritized guideline concordant care. Prior studies have reported mixed acceptance of guidelines,^18^ highlighting greater acceptance among trainees compared to their supervisors, who considered guidelines as “a threat to their professional autonomy.”^19^ The regard for guidelines in our study may reflect an increased acceptance over time or previous work done in our institution to gain clinician confidence. As we discussed guideline-concordant care with clinicians, the themes of consistency of management, access to information, and the importance of relationship and trust emerged.

We found that primary care was unique in its ability to provide relational and informational continuity, domains of continuity that do not reliably exist in urgent- and retail-care settings. The absence of informational and relational continuity was perceived as affecting the ability to provide guideline concordant care. The third domain of continuity (management continuity, or consistency of practice) was valued across primary and non–primary-care settings.

Management continuity was perceived as an outcome of providing guideline concordant care. Management continuity may also indirectly affect the ability to provide future guideline concordant care by shaping parental expectations. Both physicians and APPs across settings identified the value of management continuity, although it was less commonly described by clinicians in the walk-in clinic. The emphasis on management continuity may have been due to the strong representation from non–primary-care settings which do not have informational and relational continuity on which to rely.

The strong regard for guidelines may reflect the interdependent nature of practice in non–primary-care settings. Further, non–primary-care settings often lack relational and informational continuity, elevating the importance of management continuity. These findings indicate that establishing consistent guideline implementation and utilization throughout our healthcare system may be an important next step for an outpatient antibiotic stewardship program.

In evaluating prescribing influences, we hypothesized that clinicians would cite social determinants of health as a major factor. Although clinicians described various social determinants of health as impacting prescribing behavior, none of these factors were described as influencing prescribing and they were not described as primary drivers for the decision to prescribe. However, the behavioral feature of parental expectations for antibiotics emerged as a dominant patient-level factor.

The effect of parental expectations for antibiotics on guideline-concordant prescribing was a dominant theme in our study. The literature is mixed on whether and to what extent parents expect antibiotics at a sick visit.^12,20–24^ Regardless of actual expectations, which are often inaccurately perceived, mere perception of expectations for antibiotics has been shown to affect prescribing.^12,25^ Clinicians in our study felt that parental expectations can be positively shaped over time through consistent practice and positive, reinforced messages.

Although our findings need to be validated in additional populations, they point to broader system-level interventions that may provide local impact. We have outlined potential actions (Table 4) at each system level, from clinician and clinic to outpatient antimicrobial stewardship program, medical center and at the level of governmental public health, based on the themes (Fig. 1 and Table 3). Individuals and entities at all levels may find actions they can institute based on these findings. Instituting a combination of interventions is likely more effective than any one intervention alone.^26^

**Table 3.** Description of Major Themes, Subthemes, and Representative Quotes

Themes and Sub-Themes		Representative Quote
**Theme 1** Guidelines: Clinicians value guidelines when making decisions about prescribing antibiotics for children.		“It’s a lot easier to explain to a parent or a patient, that these are the guidelines, and you have something concrete that you can kind of show them. As a mid-level, sometimes you need something to back you up versus having the MD or DO. If I’m able to say what the guidelines are and show parents, show patients, I feel like that goes a lot further than me saying, ‘Well, this is what I recommend.’” (APP, retail clinic)
**Theme 2** Management continuity (consistency): clinicians across settings identify the importance of standardized practice within their setting and across settings, both for children and their adult parents/guardians		“There’s just a lack of consistency. That’s, I think, nationwide because we have families who come from elsewhere and they’re like, “But our doctor used to always prescribe this when this happened, why aren’t you doing that?” People don’t get a sense of what’s the right way to do things.” (Physician, primary care)
	a. Patient or parent expectations for antibiotics are a major driver impacting ability to prescribe in accordance with guidelines	“Yeah, we will often have parents explicitly ask for an antibiotic and if I don’t think that an antibiotic is indicated, I will not prescribe one just because a parent asks for it. But if it’s kind of an ambiguous situation like maybe it’s a younger child with an otitis media and maybe we can do a SNAP prescription but the family really wants to start it right away, that would be a scenario where I would take that more into consideration. I would take the parent’s preference more into consideration.” (APP, primary care)
	b. Patient or parent expectations for antibiotics are driven by previous experiences with antibiotics	“I would think maybe sometimes some of the expectation that all sore throats are strep and always get an antibiotic. Because I do get that sometimes commonly. “Oh, my throat hurts. I have strep. Every time my throat hurts I come in and I get an antibiotic.” I hear that some from patients or families. It’s like they have a set idea of what their treatment should be before they even get to the doctor’s office to get an assessment. Sometimes maybe restating their expectations that certain illnesses we treat with antibiotics. In some scenarios we might treat differently.” (Physician, urgent care)
	c. “Slow burn” of messaging over time: a subset of clinicians recognize that moving patient expectations in a way that aligns with guideline-concordant practice can take time and does not happen in an individual encounter, yet each encounter is an opportunity to move the dial	“I think explaining to parents what you’re treating and why you’re treating stuff is really important. Because then they know what to look for, and I think it empowers them, too. And there’s sometimes when they’ll say, you know, ‘I don’t think this is strep because like you’ve said before, she has cough and a runny nose.’ And I’m like, ‘You’re right. I don’t think this is strep either with a cough and a runny nose.’ So I think giving them that information empowers them and makes them make better decisions hopefully.” (Physician, primary care)
**Theme 3** Informational continuity: having reliable access to a patient’s medical history can inform decision making and antibiotic selection. Such access does not reliably exist outside of primary care (and may not always exist in primary care if patients regularly seek care outside of their medical home)		“Since I see a lot of patients that are, their primary care physician is outside the Vanderbilt system, I have to rely a lot on what the parents tell me about the history. Were they are on an antibiotic two weeks ago for the same problem. Can the parents remember which antibiotic the kid just finished? More for like the treatment failures or when I need to change a recurrent otitis.” (Physician, urgent care)
	a. Feedback after the encounter: informational continuity extends beyond the visit. In settings where workflow is fragmented, clinicians describe not knowing whether a “safety-net antibiotic prescription (SNAP) script” was filled, whether a patient returned to care, what a urine culture showed, etc.	“And so, I think they’re having encounters where they’re getting treated with an antimicrobial more often than the corresponding population that’s coming to our primary care clinic at Vanderbilt for instance. And so, I don’t know how much of that is just, there’s not as much consequence to individual providers since they’re not the ones following up and they never get to see the outcome or the feedback of starting somebody on something over the phone, for instance.” (Physician, urgent care)
**Theme 4** Relational continuity: clinicians describe the relative ease that exists in prescribing in a guideline concordant manner when they have an existing relationship with a patient. Relational continuity does not exist reliably outside primary care.		“Just knowing that the families, knowing the people makes a big difference. Cause if you know the parents then you…. Sometimes I had people that would tell me, I don’t have money for food for my kids or I don’t have, the people here aren’t going to tell me that because they don’t know me from Adam. But there they knew that if they told me that they had a need that I could get them connected to whoever they needed to be connected to to fill that need. I’d connect them to [services] where they could get food stamps or a church or the food bank or something to be able to fill a need and they’re not going to say anything to me because they don’t know me from Adam.” (APP, retail care)
	a. Trusting the clinician: a key component of the clinician–patient relationship is the trust a patient has in their clinician.	“I really have to rely on education and being contrite, ‘I’m sorry. That is not something that will help you. That is not something I’m able to do.’ And some patients accept that and some don’t.” (Physician, urgent care)
	b. Trusting the patient: similarly, clinicians trust of a patient impacts the decision to prescribe antibiotics based on concern for health literacy, compliance, and ability to follow up.	“I mean, unfortunately, I have come across way too many children who are from dual family households where they’re going to the other parent’s house for the weekend, and the other parent is not as reliable about treating, giving medicine, anything like that. If that was the situation, and it was a Tuesday, I probably would say, let’s put them on a z-pack, where I can get at least three out of five doses in the child. And hope that the father, or the other parent, would give the fifth dose. If it’s a situation where both parents are in the house, but they have very erratic work schedules, and they’ve had a history of maybe somebody didn’t write down, well, okay, I gave Motrin at 4:00, they’re not needed again till 10:00, and there’s a risk of double dosing, I would do cefdinir once a day.” (APP, urgent care)

**Table 4.** Recommended Actions Based on Identified Themes by Healthcare System Level of Intervention

Healthcare Level	Recommended Actions	Theme
Clinician	What you do in each encounter matters! Continue promoting appropriate use of antibiotics at each encounter. Look for opportunities to advance the message even if it does not result in the desired outcome during the encounter itself (similar to smoking cessation)^28^	“Slow burn” of messaging over time
	Communication training may help provide increased confidence, engagement, and parent satisfaction^29^	Parental expectations, “Slow burn” of messaging over time
Clinic	Ensure guidelines/other resources accessible in clinic	Management continuity
	Post public antibiotic commitment posters^30^	Management continuity, parental expectations
	Create opportunities for clinicians to come together, discuss priorities, and solve problems	Management continuity
Outpatient antimicrobial stewardship program	Assess clinician acceptance of guidelines and seek to improve buy-in if acceptance is lacking	Providing guideline concordant care
	Distribute easily accessible clinical practice guidelines or common outpatient complaints that can be used across settings; ensure familiarity with CPGs (education)^31^	Management continuity
	Facilitate management continuity though audit, feedback, & peer comparison on prescribing appropriateness for individual clinicians, clinics, and across medical center^31^	Management continuity
	Offer communication training^29^	Trust
	Facilitate (back-end) informational continuity (eg, feedback on outcomes of delayed prescribing and complications, or lack thereof, of conservative management)	Informational continuity
Medical center	Offer opportunities for sharing best practices and experiences across clinic settings	Management continuity
	Engage with other regional medical centers/entities to increase consistency across settings	Management continuity
	Human resources for patient follow up, eg, patient navigator^32^	Informational continuity, relational continuity
Regional, state, & local public health entities	Messaging outside of encounters, “mass media campaigns”^33^	Parental expectations
	Seek novel approaches to enhance informational continuity (eg, health information exchange,^34,35^ prescription drug monitoring program (PDMP)–like^36^ platform for antibiotics)	Informational continuity
	Bring together regional medical centers, community partners, and unaffiliated stakeholders	Management continuity
	Provide prescribing feedback for medical centers, regional entities, and community	Management continuity
	Facilitate integrated information systems, or records that travel with the patient (portable health record)^37^	Informational continuity
	Facilitate building trust and relationship: Incentivize (non)prescribing that is aligned with stewardship goals^38^, payment reform to facilitate time and trust-building (as opposed to restricted reimbursement, reimbursement penalties)^39^	Relational continuity, trust

This study is unique in the breadth of clinicians included with strong representation of APPs; however, this study had several limitations. All participants included in this study were affiliated with a single academic medical center, and the results may not be generalizable to community settings. However, clinicians who work in the walk-in and retail clinics function similarly to private practice. Additionally, antibiotic prescribing varies widely from region to region within the United States.^7^ The nonclinical factors that make up the difference between high- and low-prescribing regions likely also vary in type and degree of importance among different regions. Nevertheless, many of the overriding themes identified here are likely applicable to other regions. However, with any quality improvement or implementation project, intervention design and implementation should be grounded in local culture.^27^ Additionally, interviews were coded by a single researcher.

In conclusion, both physicians and APPs placed a high value on providing guideline-concordant care across primary and urgent- or retail-care settings; however, many patient-, clinician-, and environmental-level factors facilitated or impeded their ability to do so, including parental expectations for antibiotics. Providing guideline-concordant care was perceived to be largely driven by the presence or absence of continuity across all domains including relational, informational, and management continuity. Clinicians perceived expectations for antibiotics to be modifiable, primarily through consistent management practices. As healthcare systems become more complex and fragmented, and access to relational and informational continuity decreases, greater emphasis should be placed on management continuity.

## Supplementary material

For supplementary material accompanying this paper visit https://doi.org/10.1017/ash.2022.224. click here to view supplementary material
