# Supplementary material for: A qualitative assessment of nonclinical drivers of pediatric outpatient antibiotic prescribing: The importance of continuity
Source: Antimicrob Steward Healthc Epidemiol. 2022 Jun 29;2(1):e107. doi: 10.1017/ash.2022.224 (PMC9726583; doi:10.1017/ash.2022.224)
Supplement: Supplementary file 1 [file S2732494X22002248sup001.docx]

**Supplemental Information:**

*Semi-structured interview guide*

There are many considerations that factor into whether to prescribe an antibiotic and what antibiotic to prescribe. In addition to clinical factors such as the most likely diagnosis and the patient’s medical history, there are often other non-clinical factors to consider, for example: a patient’s preferences for treatment; their health literacy; or their ability to follow up.

1. In general, how do you choose an empiric antibiotic? What factors do you consider?
2. Are there any patient factors *unrelated* to the patient’s condition – such as social, behavioral, or environmental factors – that affect your decision to prescribe an antibiotic, or that affect the choice of antibiotic?

Now I am going to give you a set of cards that each have a different factor you might consider when prescribing an antibiotic. I would like you to place them in order from most important to least important based on your priorities when prescribing an antibiotic for a pediatric patient.

- Ease of dosing (for example, once daily dosing)
- Antibiotic spectrum (i.e. narrow vs broad)
- Guideline recommended
- Antibiotic cost/prescription coverage
- Familiarity with the antibiotic
- Antibiogram data (information about antibiotic susceptibility in your clinic, system, or community)

(Probe: tell me about how you’re thinking about this.)

1. What kind of clinical scenarios or patient factors require you to prescribe in a way that doesn’t match your priorities?
2. Tell me why this (insert first choice) is the most important factor to you when prescribing an antibiotic. (If #1 is guidelines, see sub-question under #5)
3. (If guidelines NOT #1): You ranked guideline recommended (insert rank order); tell me about why you rank guidelines where you did. How do you use guidelines?

- What about a patient or the clinical situation makes you choose an antibiotic alternative to the first line in a given situation?

1. You ranked antibiotic spectrum (insert rank order). What circumstances or kinds of cases lead you to prescribe a broader spectrum antibiotic than you think is necessary clinically?
2. How often does antimicrobial resistance affect your patients? What experiences have you had with antimicrobial resistant organisms?

Next I would like to tell you about a case. Please think about what you would do for this patient and how you would counsel them. A 5 year old child comes in to the clinic. Mom reports the patient has a history of acute otitis media including an infection treated 2 months ago. She is otherwise healthy. She now has had fever up to 101’F for 24 hours associated with complaining of ear pain on the right. Her right ear is pink with mild bulging and her left ear is clear.

(Probe: what is your thought process? What factors are are you considering?)

Tell me what you think about this case. What is your plan for the patient?

The AAP guidelines on acute otits media support watchful waiting.

Out of 10 cases of acute otitis media, how often do you try watchful waiting?

When recommending watchful waiting, do you give parents a script for delayed filling, ask them to call the clinic back, return to the clinic, or do you do something else?

What do you tell parents when you recommend watchful waiting? Do you have a counseling script or “spiel”?

What do you think is most effective in helping parents understand the role of antibiotics when you are recommending watchful waiting?

Now I would like to tell you about another case. Please think about what you would do for this patient and how you would counsel them. A 28-month-old child is brought into clinic. Someone in her daycare was diagnosed with Strep throat last week. The patient now has a fever to 102’F, is rubbing her throat, and is not wanting to eat as much as usual. Her mom tells you she suspects her daughter also has Strep throat. On review of systems, mom notes that her nose is runny as always and she has an occasional cough. The staff member who roomed the patient obtained a rapid strep before you saw the patient and it is positive. On exam, the child is shy, has a runny nose, and her throat is erythematous. She coughs 2-3 times while you are in the room.

1. Tell me what you think about this case. What is your plan for the patient?
2. Tell me about the attitudes and expectations your patients and their parents have regarding antibiotics. Do you feel like they expect antibiotics when they come to you for a sick visit?

- How do they let you know that they expect antibiotics?
- Has this changed over time?

1. Tell me about the attitudes your patients and their parents have about antibiotic *overuse*. Do patients and their parents seem to perceive antibiotic overuse as a problem? What makes you think that?

- What harms from antibiotics have parents expressed concern to you about?
- Do you talk to patients/parents about antibiotic harms, and if so, what do you talk to them about?

This next set of questions is about the setting in which you work and factors that may be unique in this environment.

1. What prescribing considerations are unique in this setting? How do they differ from other settings, for example primary care, urgent care, etc?
2. Do you think your clinic/setting is a high or low prescribing setting and what makes you think so?
3. Do you think there is a relationship between antibiotic prescribing and patient satisfaction and what is that relationship?
4. How can you and your clinic impact the problem of antibiotic overuse?
5. Do you know how your prescribing patterns compare to your peers? Are you given feedback about your prescribing patterns?

- Would it benefit you to know? Would it change your practice?

1. What are barriers to improving antibiotic prescribing?
2. What other resources could help you and your clinic improve antibiotic prescribing?

Thank you for taking the time to share your experiences. Before we’re done, I would just like to ask, is there anything else you would like to share that we haven’t talked about with regards to antibiotic use, overuse, or appropriateness; antimicrobial resistance; or antimicrobial stewardship?
